# Autologous chondrocyte implantation combined with anterior cruciate ligament reconstruction: similar short-term results in comparison with isolated cartilage repair in ligament intact joints

**DOI:** 10.1007/s00167-021-06764-9

**Published:** 2021-10-09

**Authors:** Julian Mehl, Matthias Feucht, Andrea Achtnich, Andreas B. Imhoff, Philipp Niemeyer, Peter Angele, Wolfgang Zinser, Gunter Spahn, Ingo Loer, Heino Kniffler, Gunnar Schauf, Andreas Schmitt

**Affiliations:** 1 Department of Orthopaedic Sports Medicine, Klinikum rechts der Isar, Technical University, Ismaninger Strasse 22, 81675 Munich, Germany; 2 Department of Orthopaedic Surgery, Paulinenhilfe, Diakonieklinikum, Stuttgart, Germany; 3 OCM Clinic, Munich, Germany; 4 Department for Traumatology, University Hospital Regensburg, Regensburg, Germany; 5 Department of Orthopaedic Surgery and Traumatology, St. Vinzenz Hospital, Dinslaken, Germany; 6 Center of Trauma and Orthopaedic Surgery Eisenach, Jena University Hospital, Eisenach, Germany; 7 Orthopaedie in Essen, Essen, Germany; 8 Orthopaedische Praxisklinik, Kelkheim, Germany; 9 Gelenkzentrum Bergischland, Remscheid, Germany

**Keywords:** Knee, Cartilage repair, Autologous chondrocyte implantation, Ligament, ACL

## Abstract

**Purpose:**

Both acute ruptures of the anterior cruciate ligament (ACL) as well as chronic ACL insufficiency show a high association with focal cartilage defects of the knee. However, the results after combined ACL reconstruction and cartilage repair are not well investigated. The aim of the present study was to investigate the short-term outcomes after autologous chondrocyte implantation (ACI) in combination with ACL reconstruction and to compare the results with patients who underwent isolated ACI in ligament intact knees.

**Methods:**

All patients who were registered in the German Cartilage Registry with ACI for focal cartilage defects in the knee joint in combination with ACL reconstruction and who completed the 24 month follow-up were included in the study group. A matched-pair procedure according to gender, defect location, defect size, and age was used to create a control group of patients with isolated ACI in ACL intact joints. The Knee Injury and Osteoarthritis Outcome Score (KOOS) and the numeric analog scale for pain (NAS) were used to assess the preoperative state as well as the clinical outcomes 12 and 24 months after surgery.

**Results:**

A total of 34 patients were included in both the study group (age mean 33.3 ± SD 8.8 years) and the control group (33.6 ± 8.4 years) with a median defect size of 466 (25%-75% IQR 375–600) mm^2^ and 425 (IQR 375–600) mm^2^, respectively. In comparison with the preoperative state (median 67, IQR 52–75), the study group showed a significant increase of the total KOOS after 12 months (78, IQR 70–86; *p* = 0.014) and after 24 months (81, IQR 70–84; *p* = 0.001). The NAS for pain did not change significantly in the postoperative course. In comparison with the control group there was no significant difference for the total KOOS neither preoperative (control group median 67, IQR 52–73) nor at any postoperative time point (12 months: 82, IQR 67–93; 24 months: 81, IQR 71–91).

**Conclusion:**

The clinical short-term outcomes after ACI at the knee joint in combination with ACL reconstruction are good and similar to the results after isolated ACI in ligament intact knees.

**Level of evidence:**

III.

## Introduction

Focal cartilage defects in the knee are a frequent problem among young and active patients causing pain, swelling, and impaired joint function [14]. Additionally, there is evidence that these lesions can progress over time and lead to early osteoarthritis [6, 29, 30]. Several high level studies have demonstrated that autologous chondrocyte implantation (ACI) is an effective and reliable surgical long-term option for the regeneration of articular cartilage in the knee joint [1, 15, 19, 21, 22]. However, recent literature has also demonstrated that analysis and, if applicable, therapy of possible co-factors are crucial for good postoperative results. Accordingly, the importance of the coronal leg axis for the treatment of defects at the femoral condyles and the importance of patellofemoral alignment for the patellofemoral location have been emphasized [3, 5, 11, 32].

A further co-factor which is highly associated with cartilage lesions in young and active patients is rupture of the anterior cruciate ligament (ACL) [28]. The typical injury mechanism is characterized by pivoting moments of the knee joint which leads to high shear forces on the articular surface and may result in concomitant traumatic cartilage lesions [13]. Additionally, insufficiency of the ACL leads to chronic anterior and rotational instability and is a known risk factor for the development of secondary posttraumatic cartilage injuries [18, 24, 25].

Although there is still no consensus about the influence of ACL reconstruction on the development of posttraumatic osteoarthritis, there is evidence that it can at least decrease the risk of focal secondary cartilage injuries [7, 10, 24]. However, the influence of ACL reconstruction on regenerative cartilage repair procedures like ACI is unclear. Filardo et al. have performed a systematic review on articles dealing with cartilage lesions detected at the time of ACL reconstruction [12]. Of the 36 included studies, 10 studies specified the performed cartilage repair techniques and only three studies investigated the combination of ACL reconstruction and ACI [2, 9, 33]. While two of these studies investigated small cohorts of 18 and 19 patients, respectively, without control groups, the study by Dhinsa et al. included 47 patients with combined ACI + ACL reconstruction in comparison with a small group of 12 patients after isolated ACI [9].

The aim of the present study was to evaluate the postoperative outcomes after ACI of the knee joint in combination with ACL reconstruction in a representative cohort. Additionally, a matched-pair analysis was performed to compare the results with patients who underwent ACI alone in ligament intact knees.

It was hypothesized that the combination of ACI and ACL reconstruction leads to good postoperative outcomes similar to the results following ACI alone in ligament intact knees.

## Materials and methods

### The German cartilage registry

The German Cartilage Registry (KnorpelRegister DGOU) is a multicenter registry in Germany, Switzerland, and Austria, which was introduced in October 2013 and aims on evaluating patients who underwent treatment of focal cartilage defects [20]. Data were collected by means of a web-based Remote Data Entry System and was recorded by both the physicians and the patients. The registry is conducted in accordance with the 1964 Declaration of Helsinki and registered at germanctr.de (DRKS00005617). The registration of data was approved by the local ethics committees of every participating institution. Primary approval was given by the ethics committee at the University of Freiburg (No. 520/14). Informed consent was obtained from all individual participants included in the study.

### Study population

All patients who were registered in the German Cartilage Registry from October 2013 until December 2020 with ACI for focal cartilage defects in the knee joint in combination with ACL reconstruction and who completed the 24 month follow-up were included in the study group. Patients with a follow-up of less than 24 months and patients with further concomitant surgical procedures were excluded from the study group.

A control group was established by means of a matched-pair procedure. Therefore, all patients who were registered in the German Cartilage Registry from October 2013 until December 2020 because of isolated third- or fourth-generation ACI for focal, traumatic, or posttraumatic cartilage defects in ACL intact knee joints were analyzed. From this cohort, the best matching patient according to gender, defect location, defect size, and age was assigned to each patient of the study group. Patients with degenerative cartilage defects (no trauma association), patients with further concomitant surgical procedures, and patients with a follow-up of less than 24 months were excluded from the control group.

### Data acquisition

The patient’s demographic data, information about the present pathology, and about the performed surgical treatment were taken from the baseline data of the German Cartilage registry. Regarding the etiology of the cartilage defects, degenerative defects and traumatic defects were differentiated. Trauma was defined as sudden, unexpected event causing damage to the knee joint. The duration of symptoms was given in months. The morphologic characteristics of the cartilage defects were recorded by the surgeon based on the intraoperative findings. The defect size was determined using its maximum width and length in millimeter (mm). To make the size comparable, both values were multiplied and the final value was given in mm^2^. The severity of the cartilage defect was classified into four grades according to the ICRS classification [4]. The location of the defects was described based on the following sites: patella, trochlea, medial femoral condyle, lateral femoral condyle, medial tibia plateau, and lateral tibia plateau.

The Knee injury and Osteoarthritis Outcome Score (KOOS) [27] was used as standardized and validated patient-reported outcome score to assess the preoperative state as well as the clinical outcomes 12 and 24 months after surgery [26]. The results were given for the total KOOS and separately for the five subscores: KOOS-Activities of Daily Living (KOOS-ADL), KOOS-Pain, KOOS-Quality of Life (KOOS-QOL), KOOS-Symptoms, and KOOS-Sports. Additionally, the numeric analog scale (NAS) for pain and a questionnaire regarding satisfaction with the postoperative results were used for further assessment at the same time points.

### Statistical analysis

Statistical analysis was conducted with IBM SPSS Statistics version 26 (IBM Corp., Armonk, NY, USA). Normal distribution of quantitative variables was examined and graphically confirmed with the Shapiro–Wilk normality test. Normally distributed data were represented as mean ± standard deviation (SD) and not normally distributed data were represented as median and 25–75% interquartile range (IQR). Qualitative variables were represented as absolute and relative frequencies. The clinical outcome scores were compared with the preoperative state separately for both study groups using the paired t test for normally distributed data and the Wilcoxon test for not normally distributed data. Between-group comparisons of quantitative data were also performed using the paired t test for normally distributed data and the Wilcoxon test for not normally distributed data. Qualitative data were compared between the groups using the Chi-square test and Fisher’s exact test. The alpha level for all analyses was set at 5%.

Sample size calculation was performed using the software G*Power 3.1 (HHU Düsseldorf, Germany). It was based on the results of a recent prospective clinical trial which investigated the clinical outcomes 24 months after matrix-associated autologous chondrocyte implantation [19]. A sample size of 30 patients per group was necessary to detect a difference of 8 points in the total KOOS with a power of 80% and at an *α* level of 0.05.

## Results

**Fig. 1:**
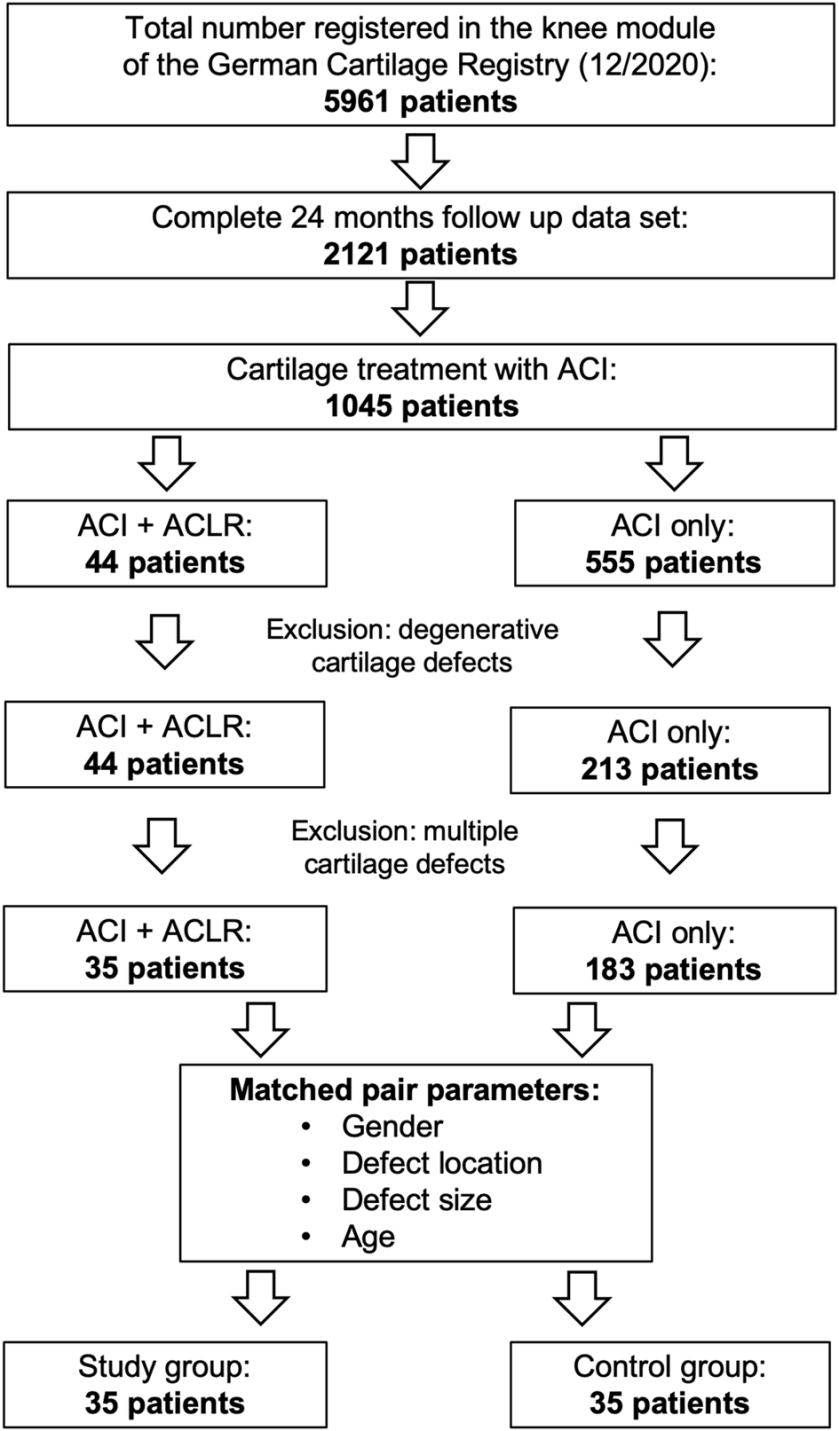
Selection process of included patients. *ACI* autologous cartilage implantation, *ACL* anterior cruciate ligament, *FU* follow-up

A total of 34 patients were included in the study group and 34 further patients were included in the matched control group (Fig. 1). There were no significant group differences regarding the pre- and intraoperative characteristics (Table 1).

**Table 1:** Pre- and intraoperative patient characteristics

	Study group	Control group	*p* value
Gender (f/m)	8/26	8/26	n.s.
Age (years)	33.3 ± 8.8	33.6 ± 8.4	n.s.
BMI (kg/m^2^)	26.2 ± 3.5	26.5 ± 3.5	n.s.
Duration of symptoms (months)	6 (IQR 3–18)	8 (IQR 6–12)	n.s.
Defect location			
FC med	19 (56%)	19 (56%)	n.s.
FC lat	10 (29%)	10 (29%)	
Patella	4 (12%)	4 (12%)	
Trochlea	1 (3%)	1 (3%)	
Defect size (mm^2^) Defect size (mm^2^)	466 (IQR 375–600)	425 (IQR 375–600)	n.s.
Defect grade (ICRS)			
Grade 3	19 (56%)	13 (35%)	n.s.
Grade 4	15 (44%)	21 (62%)	

Normally distributed data is given as mean ± standard deviation. Not normally distributed data are given as median (25%-75% IQR)

f, female; m, male; n.s., not significant; BMI, body mass index; FC, femoral condyle; med., medial; lat., lateral

**Fig. 2:**
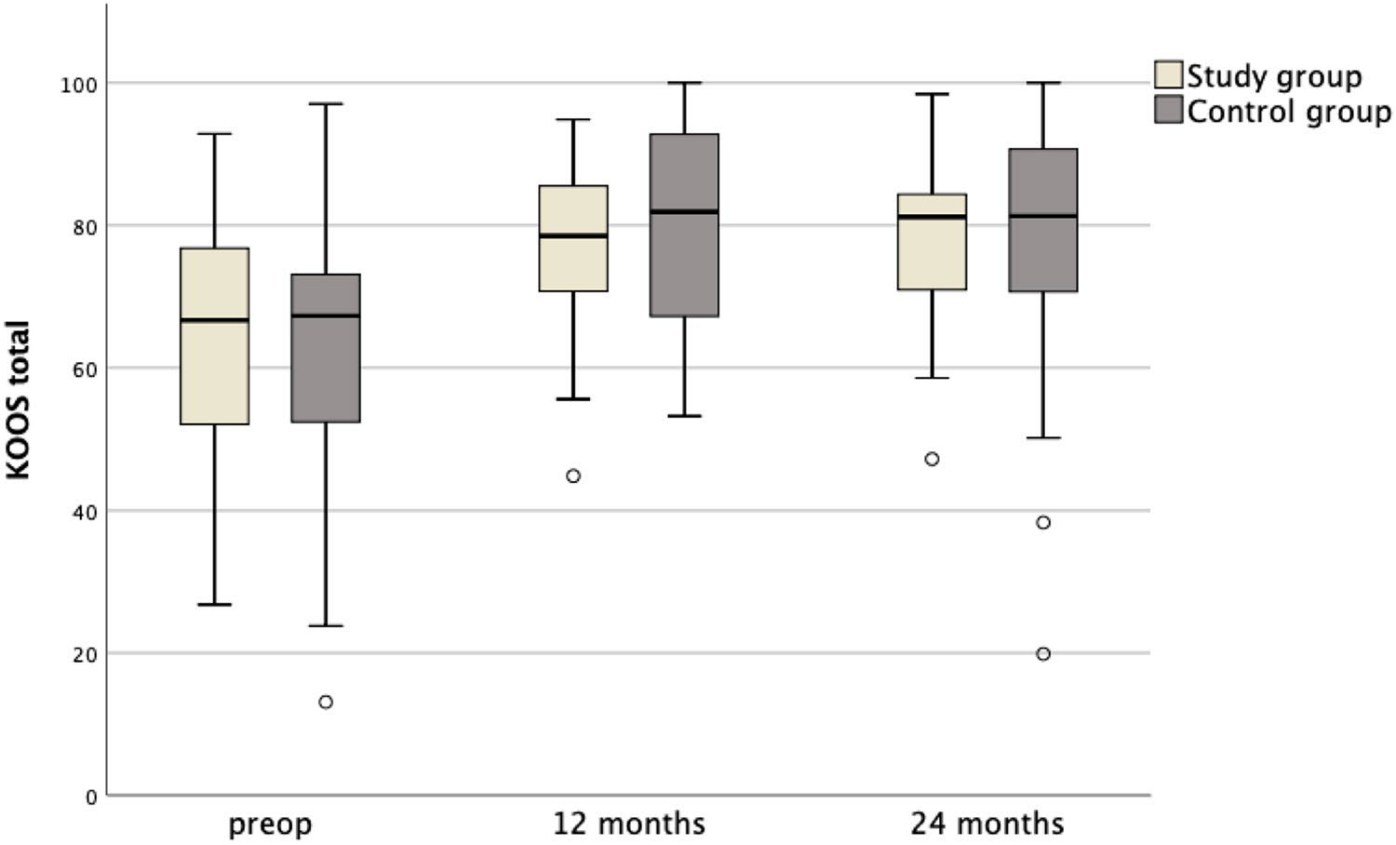
Box plots representing the total KOOS values in comparison between both groups preoperatively as well as 12 months and 24 months after surgery

In comparison with the preoperative state, the patients in the study group showed a significant improvement of the total KOOS after 12 months and after 24 months (Table 2, Fig. 2). Regarding the subscores, there were significant improvements of the KOOS-ADL, the KOOS-QOL, and the KOOS-Sports after 24 months. The KOOS-Pain and the KOOS-Symptoms did not demonstrate significant improvements after 24 months (Table 2).

**Table 2:** Results of the KOOS and NAS

	Study group		Control group		*p* value^#^
	Median (IQR)	*p* value*	Median (IQR)	*p* value*	
Total KOOS					
Preop	67 (52–75)	*p* = 0.001	67 (52–73)	*p* < 0.001	n.s.
24 months	81 (70–84)		81 (71–91)		n.s.
KOOS-ADL					
Preop	80 (66–92)	*p* = 0.006	81 (64–91)	*p* = 0.004	n.s.
24 months	90 (85–96)		92 (78–99)		n.s.
KOOS-Pain					
Preop	78 (61–86)	n.s.	69 (47–75)	*p* = 0.001	n.s.
24 months	81 (69–86)		86 (68–92)		n.s.
KOOS-QOL					
Preop	31 (19–39)	*p* < 0.001	38 (22–44)	*p* < 0.001	n.s.
24 months	44 (34–63)		56 (31–72)		n.s.
KOOS-Symptoms					
Preop	68 (54–79)	n.s.	64 (54–75)	*p* = 0.010	n.s.
24 months	68 (63–82)		75 (61–89)		n.s.
KOOS-Sports					
Preop	30 (4–55)	*p* = 0.003	35 (18–55)	*p* < 0.001	n.s.
24 months	60 (35–78)		65 (43–80)		n.s.
NAS					
Preop	2 (1–3)	n.s.	3 (1–6)	n.s.	n.s.
24 months	2 (1–4)		2 (1–4)		n.s.

Results given as median (25–75% IQR)

Sig.*, significance for intra-group comparison between preoperative and 24 month follow-up; Sig.^#^, significance for inter-group comparison for each score preoperative and at 24 month follow-up; preop, preoperative; n.s., not significant

The NAS did not change significantly in the postoperative course. Two patients needed revision surgery due to postoperative complications. One patient needed removal of a loose interference screw and one patient needed arthrolysis due to postoperative arthrofibrosis. At the 24 month follow-up, 3 patients (8.8%) were “unsatisfied” with the postoperative result (Fig. 4).

In the control group, there was a significant improvement of the total KOOS in comparison with the preoperative state after 12 months and after 24 months (Table 2, Fig. 2). All subscores of the KOOS showed a significant improvement after 24 months (Table 2).

The NAS did not change significantly in the postoperative course. Two patients needed revision surgery due to failure of the ACI procedure. One patient was subsequently treated by osteochondral transplantation and one patient was treated by microfracturing. Only one patient (2.9%) was “unsatisfied” with the postoperative result 24 months after surgery (Fig. 4).

**Fig. 3:**
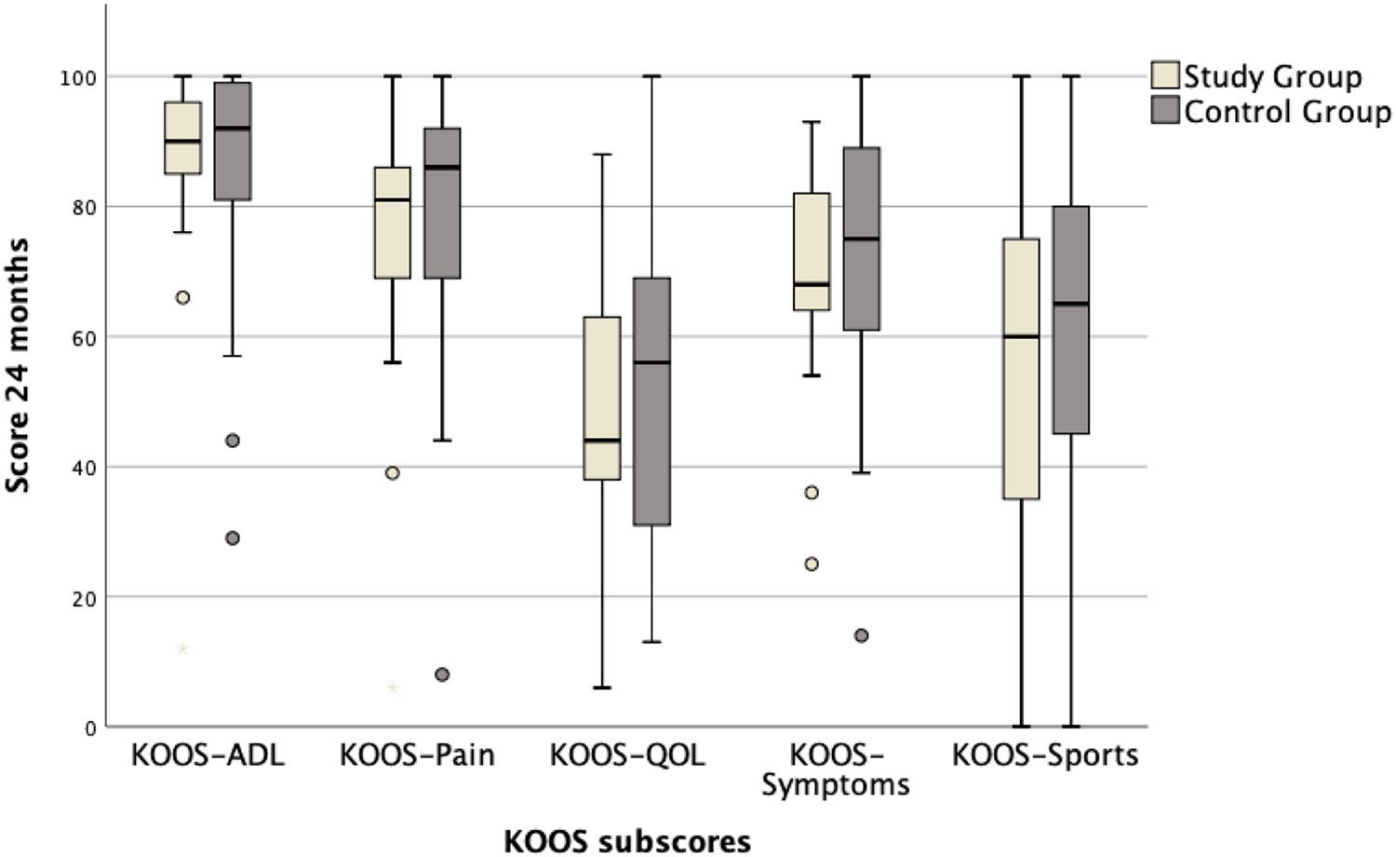
Box plots representing the values of the KOOS subscales 24 months after surgery in comparison between both groups

**Fig. 4:**
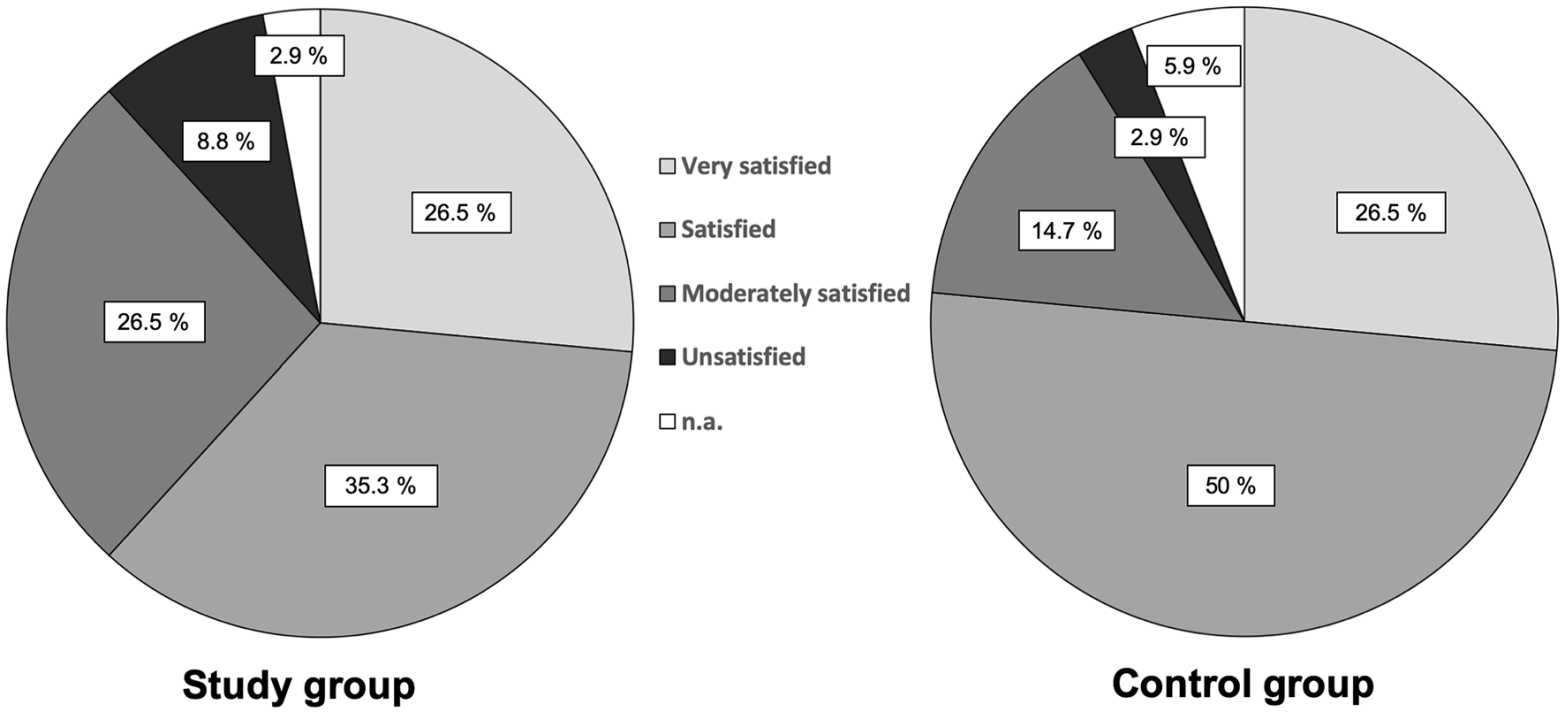
Pie charts representing the patients’ satisfaction with the postoperative results 24 months after surgery in comparison between both groups

The group comparison of the total KOOS, of the KOOS subscores and of the NAS did not show any significant differences neither preoperatively nor at any postoperative time point (Table 2, Fig. 3). There was also no significant group difference regarding the postoperative satisfaction rates (Fig. 4).

## Discussion

The main finding of the present study was that ACI at the knee joint in combination with ACL reconstruction led to good clinical short-term results with significant improvement in comparison with the preoperative status. Furthermore, the postoperative outcome did not show significant differences in comparison with isolated ACI in ligament intact knee joints.

The main goal of the present study was to investigate the influence of an additional ACL reconstruction on the clinical outcomes after ACI in the knee joint. For this purpose, the German Cartilage Registry was used, and following strict inclusion and exclusion criteria, a homogeneous study group was built and compared with a control group of ligament intact knee joints according to a matched-pair study design.

The study group showed a significant improvement of the total KOOS after 12 months and 24 months in comparison with the preoperative state, while the improvement after 6 months did not reach statistical significance. Only a few previous studies have investigated the clinical outcome after combined ACL reconstruction and regenerative cartilage procedures. Good clinical outcomes have been reported after combined ACL reconstruction and osteochondral autograft transplantation (OCT) [17, 31, 34]. Additionally, Tirico et al. and Wang et al. did not find significant differences of the postoperative outcomes in comparison with isolated OCT [31, 34]. In 2017 Pike et al. published a case series of 26 patients undergoing ACL reconstruction in combination with ACI for large cartilage defects with an average size of 8.4 cm^2^ [23]. After a mean follow-up of 95 months, the authors found significant improvements of all outcome measures, while they also reported a failure rate of 31%. Similar results were published by van Duijvenbode et al. [33] in 2015, who investigated 19 patients undergoing ACI after previous ACL reconstruction with a median time between ACL reconstruction and ACI of 52 months (range 16–369 months). In comparison with the status before ACI, a significant improvement of the IKDC score and the KOOS-quality-of-life subscore was detected after 24 months, while no significant improvement was seen for any other KOOS subscore. Additionally, 37% of the patients needed revision surgery within the first 2 years. Both of these trials were cohort studies without a control group. Therefore conclusions regarding the influence of the additional ACL reconstruction were limited.

A comparative study was conducted by Dhinsa et al. [9] investigating three independent groups of patients with previous ACL injury undergoing ACI at the knee. The first group (22 patients) was treated with simultaneous ACI and ACL reconstruction, the second group (25 patients) was treated with ACL reconstruction first and after an average of 74 months later with ACI, and the third group (12 patients) was treated with ACI alone, because the injured ACL was felt to be clinically stable. The authors found satisfying postoperative results for both groups with additional ACL reconstruction after a mean follow-up of more than 5 years. However, the group with ACI alone was found to have significantly better outcomes. The main methodical flaws of this study were the small sample size of the control group and the lack of completely ligament intact knees as controls.

In the present study, a matched-pair control group of ligament intact knees was chosen to investigate the role of an additional ACL reconstruction as possible negative influence factor on the outcome after ACI in the knee joint. Before surgery, both groups showed similar values for the KOOS and the NAS and there were also no statistical significant differences at any of the postoperative time points. Additionally, there was no relevant group difference regarding the rate of complications or revision surgery. It is well known that injuries of the ACL are relevant risk factors for the development of secondary cartilage defects already within a few months and that ACL reconstruction is not able to completely eliminate this risk [10, 24]. However, the results of present study showed that patients who underwent additional ACL reconstruction because of ACL insufficiency were able to achieve similar clinical short-term outcomes after ACI like patients with ligament intact knees.

The role of high-grade cartilage injuries in patients with ACL reconstruction has been previously investigated by Cox et al. [8] who have performed a prospective multicenter cohort study on 1307 patients after ACL reconstruction. Using a logistic regression model, the authors found that grade 3 and 4 cartilage injuries especially of the femoral condyles were significant negative predictors of the clinical outcome. A systematic review by Filardo et al. [12] including 37 studies confirmed this negative influence and also demonstrated a significantly increased risk for progression of osteoarthritis if cartilage lesions were present at the time of ACL reconstruction. This systematic review also included a few studies, which investigated the combination of ACL reconstruction and cartilage repair procedures like ACI. However, due to small study cohorts and methodical flaws like the lack of control groups, the authors were not able to draw profound conclusions regarding the benefit of additional cartilage repair in combination with ACL reconstruction. Although this question cannot be answered by the present study either, the results of the present study demonstrate that the combined procedure of ACL reconstruction and ACI for concomitant cartilage defects works well with a significant improvement of clinical scores and a low complication rate. Based on these findings and against the background that cartilage repair surgery has proven to decrease the risk for early osteoarthritis [16], in the author’s opinion, additional cartilage repair in combination with ACL reconstruction should be considered especially in young and active patients with high-grade chondral defects.

Besides certain methodical strengths, there are several limitations to the present study. First, this is a comparative cohort study based on data collected in a registry. Therefore, the general limitations of multicenter registry studies apply to this study as well, including the possible influence of multiple participating surgeons. However, in many cases, only data of large registries allow to create representative, homogeneous study cohorts to investigate specific scientific questions. Accordingly, strict inclusion and exclusion criteria were used in the present study and a matched-pair analysis was used to outweigh the relatively small sample size. However, it must be considered that a larger study cohort with a greater statistical power would have detected possible group differences more likely.

A further limitation of the present study was that the data collected in the registry were limited and some information especially regarding the medical history was missing. For example, no specific information about degenerative cartilage lesions already prior to the trauma and no information about the ACL reconstruction technique like the graft choice were available. Additionally, no postoperative clinical examinations and no postoperative radiological investigations were performed. Therefore, a possible correlation of the clinical results with knee stability or with healing of the ACI in magnetic resonance imaging could not be investigated.

Furthermore, only short-term outcomes with a follow-up of 24 months are represented by present study. A longer follow-up may lead to a higher rate of clinical failures and thus make the group comparison more conclusive.

Additionally, conclusions regarding the benefit of the ACI procedure in ACL reconstructed knees are limited based on the present study results. To answer this specific question, a further control group of patients with isolated ACL reconstruction in ACL-deficient knees with focal cartilage defects would have been necessary.

Despite these limitations, the findings of the present study are of distinct clinical relevance as ACI in the knee in combination with ACL reconstruction is a successful treatment option in patients with focal cartilage defects and ACL insufficiency. Additionally, the need for an additional ACL reconstruction does not seem to have a negative influence on the postoperative short-term outcome when compared with ACI alone in ACL intact knees.

## Conclusion

The clinical short-term outcomes after ACI at the knee joint in combination with ACL reconstruction are good and similar to the results after ACI alone in ligament intact knees.
